# Three plasma biomarkers of HTLV-1-associated myelopathy/tropical spastic paraparesis

**DOI:** 10.1186/1742-4690-8-S1-A43

**Published:** 2011-06-06

**Authors:** Paul Kirk, Aviva Witkover, Alan Courtney, Alexandra M Lewin, Robin Wait, Michael P Stumpf, Sylvia Richardson, Graham P Taylor, Charles R Bangham

**Affiliations:** 1 Centre for Bioinformatics, Division of Molecular Biosciences, Imperial College, London, SW7 2AZ, UK; 2 Department of Immunology, Wright-Fleming Institute, Imperial College, London, W2 1PG, UK; 3 Department of Clinical Biochemistry, Imperial Academic Health Sciences Centre, St Mary’s Hospital, London W2 1PG, UK; 4 Department of Epidemiology and Biostatistics, Imperial College, London, W2 1PG, UK; 5 Kennedy Institute of Rheumatology, Imperial College London, 65 Aspenlea Road, London W6 8LH, UK; 6 Department of Genitourinary Medicine and Communicable Diseases, Wright-Fleming Institute, Imperial College, London, W2 1PG, UK

## 

The pathogenesis of HAM remains uncertain: the disease is thought to be caused by the immune response to HTLV-1, possibly by bystander damage to neurons in the spinal cord. The strongest correlate of HAM in HTLV-1-infected individuals is the proviral load of HTLV-1, i.e. the percentage of peripheral blood mononuclear cells that carry the provirus. To aid in the differential diagnosis of HAM, and to search for clues as to the pathogenetic mechanisms of the disease, we carried out SELDI mass spectrometry on plasma samples from 68 HTLV-1-positive individuals, 16 uninfected controls and 11 patients with secondary progressive MS. We identified three plasma protein biomarkers that are specifically associated with HAM, independently of proviral load. The three proteins were identified by tandem mass spectrometry as b2-microglobulin, calgranulin B, and apolipoprotein A2. Using the two most strongly associated biomarkers, b2-microglobulin and calgranulin B, we derive a simple algorithm that correctly classified the disease status (presence or absence of HAM) in 81% of HTLV-1-infected subjects in the cohort.

